# Microstructural and Mechanical Characterization of Cu/SnAg Pillar Bumps with Ni-Less Surface Finish Utilizing Laser-Assisted Bonding (LAB)

**DOI:** 10.3390/ma18081834

**Published:** 2025-04-16

**Authors:** Sang-Eun Han, Dong-Gyu Choi, Seonghui Han, Tae-Young Lee, Deok-Gon Han, Hoo-Jeong Lee, Sehoon Yoo

**Affiliations:** 1 Regional Industry Innovation Department (Growth Engine), Korea Institute of Industrial Technology, Incheon 21999, Republic of Korea; sangeun35@kitech.re.kr (S.-E.H.); cdg0827@kitech.re.kr (D.-G.C.); han6755@kitech.re.kr (S.H.); 2 School of Materials Science and Engineering, Sungkyunkwan University, Suwon 16419, Republic of Korea; 3 Department of Advanced Materials Engineering, Kyonggi University, Suwon 25440, Republic of Korea; 4 School of Materials Science and Engineering, Andong National University, Andong 36729, Republic of Korea; 5 School of Materials Science and Engineering, Tech University of Korea, Siheung 15073, Republic of Korea; lty1226@tukorea.ac.kr; 6 MK Chem & Tech Co., Ltd., Ansan 15434, Republic of Korea; deokgon.han@gmail.com

**Keywords:** laser-assisted bonding (LAB), Ni-less surface finish, direct palladium immersion gold (DPIG), Cu/SnAg pillar bump, shear strength, fracture surface

## Abstract

In this study, an interconnection was formed between a Cu/SnAg pillar bump and an Ni-less surface-treated Cu pad through laser-assisted bonding (LAB), and its bonding characteristics were evaluated. The LAB process influences the bond quality and mechanical strength based on the laser irradiation time and laser power density. The growth of the intermetallic compound (IMC) in the joint cross-section was observed via FE-SEM analysis. Under optimized LAB conditions, minimal IMC growth and high bonding strength were achieved compared to conventional thermo-compression bonding (TCB) and mass reflow (MR) processes. As the laser irradiation time and laser power density increased, solder splashing was observed at bump temperatures above 300 °C. This is hypothesized to be due to the rapid temperature rise causing the flux to vaporize explosively, resulting in simultaneous solder splashing. With increasing laser power density, the failure mode transitioned from the solder to the IMC.

## 1. Introduction

Recently, the emergence of electronic megatrends such as artificial intelligence (AI), 5G communications, the internet of things, and smart cars has significantly increased the demand for high-performance semiconductors. Traditionally, the miniaturization of semiconductor transistors has driven the development of high-performance semiconductors, with Moore’s law predicting a doubling of the number of transistors on a chip approximately every two years [1]. However, as transistors shrink to the atomic scale, traditional scaling faces physical and economic limitations, necessitating innovative packaging solutions beyond conventional silicon scaling. Advanced packaging technologies, such as heterogeneous integration, have been introduced to address these challenges by integrating multiple types of semiconductors into one package, thereby improving the performance, power, area, and cost of semiconductor devices [2,3].

This trend toward heterogeneous integration in advanced packaging technology requires higher input/output (I/O) density and the development of interconnected technology that can support very fine pitch sizes [2]. The existing mass reflow (MR), the most widely used bonding technology, connects chips with self-alignment in a reflow oven. Despite its high reliability and low cost, MR has limitations in supporting the fine pitches required for heterogeneous integration [4]. Consequently, thermo-compression bonding (TCB), which connects the chip and the substrate using heat and pressure, has been applied to fine-pitch high-performance packaging. However, TCB requires long bonding times and high equipment costs, posing economic challenges for mass production [5].

To overcome these problems, laser-assisted bonding (LAB) was introduced. Unlike conventional laser soldering where the beam is focused, the laser light source in LAB uses a square-shaped spread beam that emerges to irradiate a larger area. This method enables localized heat energy to be evenly distributed, minimizing the thermal stress and suppressing warpage, making it suitable for fine-pitch applications [6]. Additionally, LAB enables fast joining and improves productivity due to the instantaneous energy transfer of the laser [7,8,9]. Therefore, LAB is emphasized as an alternative that can meet the needs of high-performance semiconductor packaging while complementing the disadvantages of MR and TCB. Recent research has explored applying LAB to various interconnections. For instance, Braganca et al. [10] used LAB and non-conductive paste (NCP) to perform the 3D stack bonding of up to six layers of Si chips, with TSVs formed. Gim et al. [11] formed a 40 µm fine-pitch bump using LAB, confirming that the solder was sufficiently wetted even in the short time of 0.5 s and that bonding was possible through self-alignment of the bump. Jang et al. [12] conducted thermomechanical analysis and actual measurements, finding that LAB reduced the warpage in flip chip packages to about one-third compared to MR, confirming LAB as an effective interconnection method for suppressing warpage.

For fine-pitch interconnection, the substrate surface finish is also crucial. The traditional electroless nickel electroless palladium immersion gold (ENEPIG) surface finish is considered reliable but is less suitable for fine-pitch applications due to the thick nickel layer [13]. Additionally, the nickel layer of ENEPIG may cause signal loss in 5G applications due to its ferromagnetic properties [14]. Consequently, direct palladium immersion gold (DPIG), an Ni-free surface finish, is being explored for fine-pitch interconnection and 5G applications. DPIG, which removes the Ni(P) layer from ENEPIG, offers much lower signal loss at high frequencies compared to conventional ENEPIG or thin ENEPIG [15]. Furthermore, the submicron thickness of the DPIG makes it suitable for fine-pitch substrates [16].

This study aimed to observe the bonding characteristics of fine-pitch Cu/SnAg pillar bumps by LAB using an Ni-less DPIG surface finish. The interfacial microstructures of the Cu/SnAg pillar bumps and DPIG surface finishes were observed under various LAB conditions and the mechanical properties, such as the shear strength and failure mode of the joint, were compared with traditional methods such as TCB. The innovation of this study lies in the novel application of LAB technology in combination with the DPIG surface finish for the first time in ultra-fine-pitch interconnections, presenting a pioneering solution that enhances the reliability and performance of fine-pitch interconnection applications.

## 2. Materials and Methods

**Figure 1:**
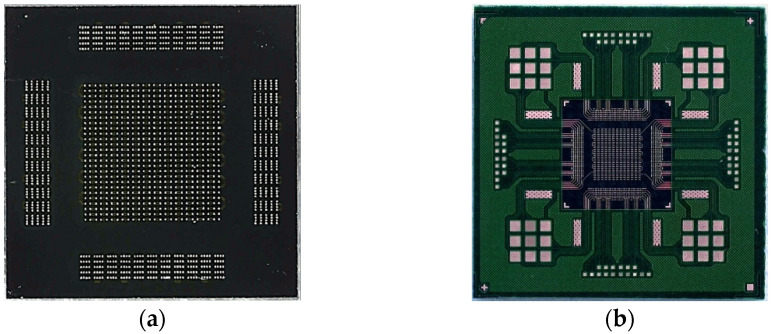
(**a**) Test chip and (**b**) test substrate in this study.

**Figure 2:**
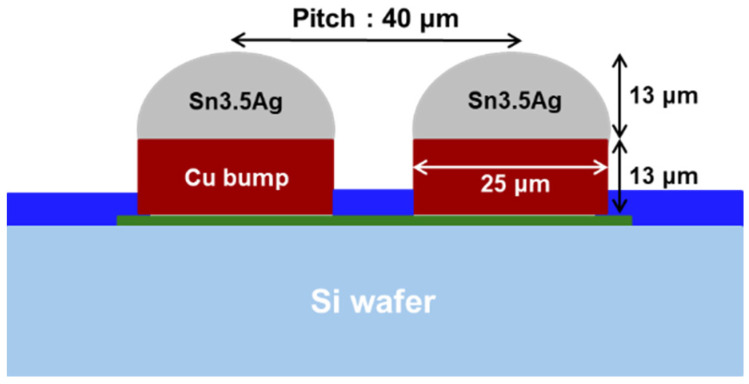
Schematic diagram of the Cu/SnAg pillar bumps.

**Figure 3:**
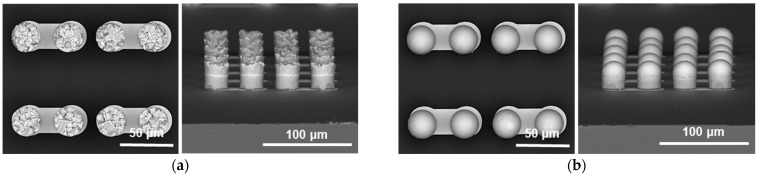
SEM micrographs of the Cu/SnAg pillar bumps (**a**) before reflow and (**b**) after reflow.

In this study, as shown in Figure 1, a silicon (Si) test chip with Cu/Sn-3.5wt%Ag (SnAg) pillar bumps and a bismaleimide triazine (BT) substrate was used. The test chip’s size was 4.4 × 4.4 mm^2^, and the substrate’s size was 12 × 12 mm^2^. The Cu/SnAg pillar bumps were fabricated on the test chip with a pitch of 40 μm (Figure 2). The diameter of the Cu/SnAg pillar bumps was 25 μm. The Cu pillar and the SnAg cap had a height of 13 μm. Before the bonding process, the Cu/SnAg pillar bumps plated on the test chip were subjected to reflow at a maximum temperature of 250 °C to transform the SnAg solder into a semi-spherical shape, which is a process used to equalize the solder composition. Images of the Cu/SnAg pillar bumps before and after reflow are shown in Figure 3.

**Figure 4:**
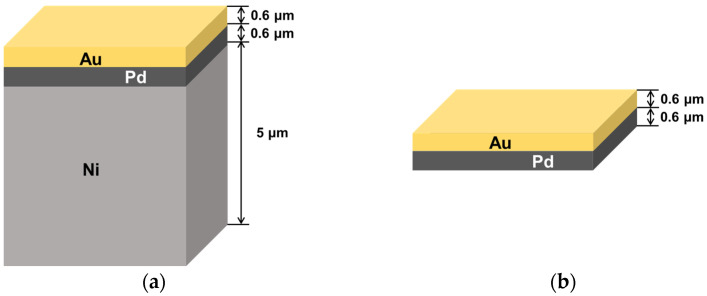
Schematic images of the (**a**) ENEPIG surface finish and (**b**) DPIG surface finish.

Figure 4 depicts the schematic diagram of the DPIG and ENEPIG surface finishes used in this study. The DPIG, which is an Ni-less surface finish, had palladium and gold layer thicknesses of 0.6 μm each, while the ENEPIG had palladium and gold layer thicknesses of 0.6 μm, and a nickel layer of 5 μm. The DPIG surface finish involved electroless palladium plating and immersion gold plating performed at 65 °C and 83 °C, respectively. The electroless palladium and immersion gold plating solutions were supplied by MK Chem & Tech (Neozen Pd-P and Flash Gold IG-10, respectively, Ansan, Republic of Korea). The comparative sample, the ENEPIG, also used MK Chem & Tech’s electroless nickel plating solution (Neozen MP-K series, Ansan, Republic of Korea) at a plating temperature of 83 °C. The palladium and gold plating processes for the ENEPIG were the same as the DPIG surface finish process.

**Figure 5:**
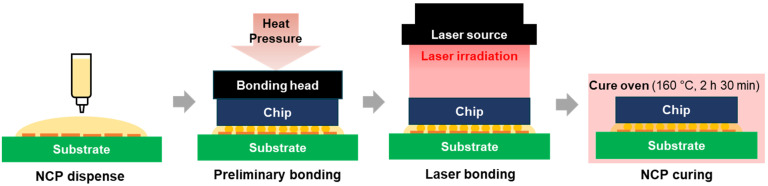
Schematic diagram of the entire process of LAB.

**Figure 6:**
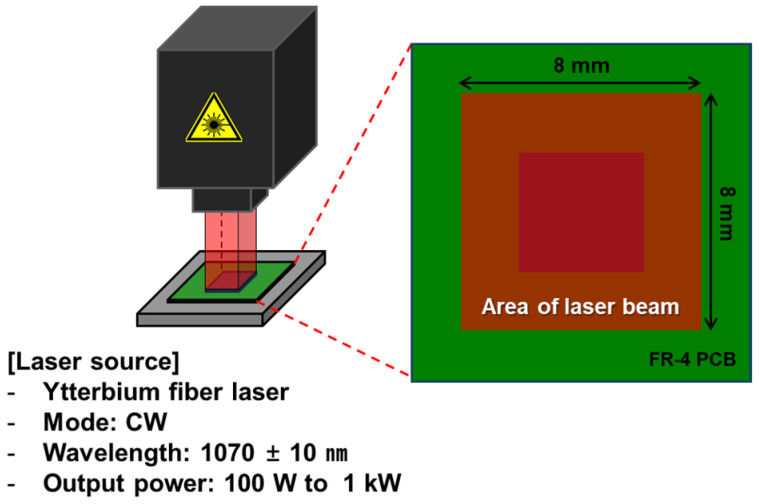
Schematic diagram of the LAB equipment.

**Figure 7:**
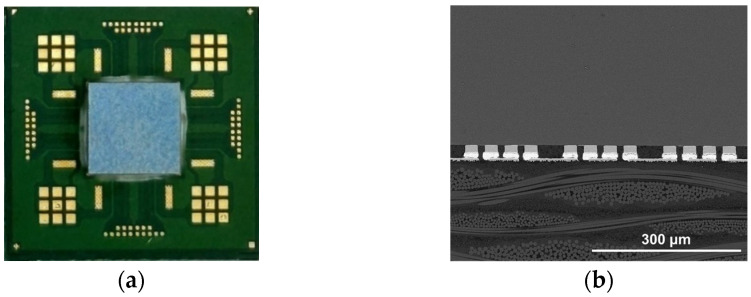
(**a**) Optical micrographs of the test sample after the entire process and (**b**) cross-sectional SEM micrograph of the bump interconnections.

LAB was used to bond the Cu/SnAg pillar bumps of the test chip to the surface-finished Cu pads of the test board. The LAB process consisted of four steps: NCP dispensing, preliminary bonding, main bonding, and NCP curing (Figure 5). First, in the NCP dispensing stage, liquid flux was applied to the Cu pad of the test board, and then NCP (NCP 5209, Henkel, Düsseldorf, Germany) was dispensed. In the next preliminary bonding step, the Cu/SnAg pillar bump and surface-finished Cu pad were aligned, and temporary bonding was performed using a flip chip bonder (NM-SB50A, Panasonic, Osaka, Japan) at 180 °C, below the melting point of SnAg solder. During temporary bonding, the SnAg solder does not melt, and the NCP hardens slightly to secure the chip. In the main bonding step, the joining was performed with an LAB machine (INYA 1000W, INLASER, Bucheon, Republic of Korea) under the conditions of a laser beam size of 8 × 8 mm^2^, laser power density of 2.52~2.81 W/mm^2^, and a time of 0.7~1.9 s. Figure 6 is a schematic diagram of the LAB equipment. After the main bonding, NCP curing was performed in an oven for 2.5 h. For comparison with the LAB process, the TCB process was also performed. The TCB process joined the chip and board at a maximum temperature of 240 °C and a pressure of 20 N. The MR process was performed by aligning at the same pre-bonding temperature as the LAB and then bonding in a vacuum environment at a maximum temperature of 250 °C. Figure 7 shows an image of the final bonded test sample.

**Figure 8:**
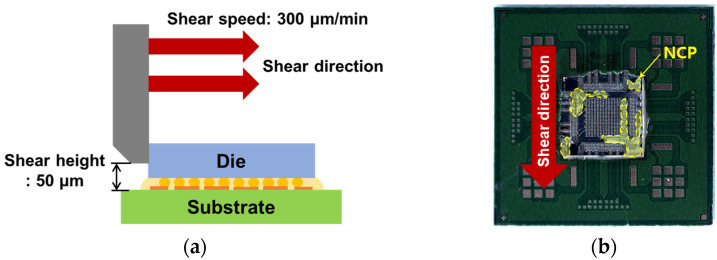
(**a**) Die shear test schematic diagram and (**b**) optical micrograph of the test sample after the shear test.

After bonding the chip and test substrate, the alignment and bump status were observed using an X-ray (XSCAN-H160-OCT, XAVIS, Seongnam, Republic of Korea). The microstructure of the joint was observed using a field emission scanning electron microscope (SEM, Inspect F, FEI, United States of America), and the composition was analyzed using energy dispersive spectroscopy (EDS, Superdry II, Thermo Fisher Scientific, Waltham, MA, USA). To evaluate the mechanical properties of the joint, a die shear test was conducted using a shear tester (Dage 4000, Nordson, Westlake, OH, USA). The conditions for the die shear test were a shear speed of 300 μm/sec and a die height of 50.0 μm (Figure 8a). After the die shear test, the image of the substrate is shown in Figure 8b, the fracture surface was observed with an SEM.

## 3. Results

### 3.1. Joint Properties with LAB Power Density

**Figure 9:**
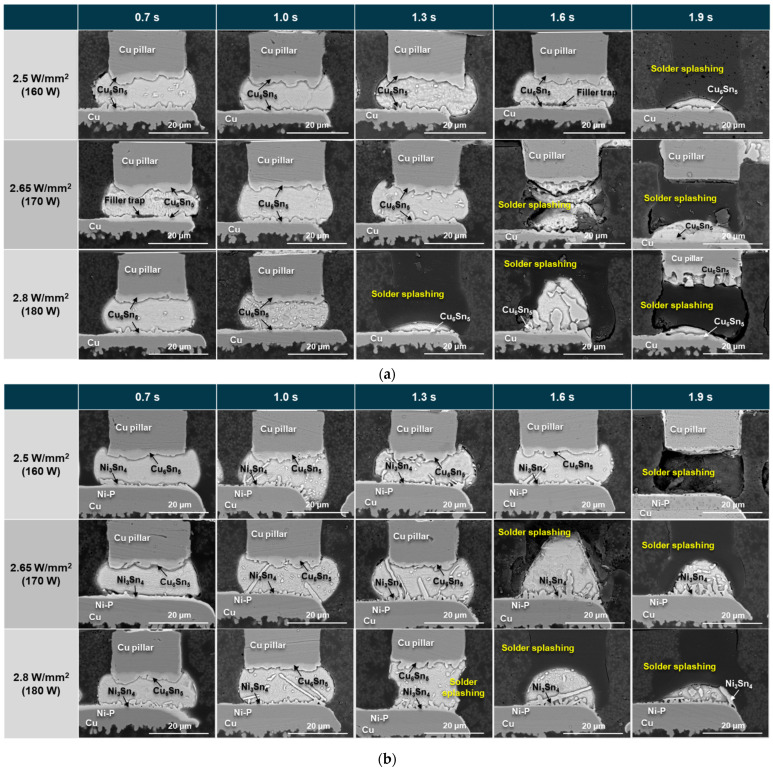
Cross-sectional micrographs of the SnAg/Cu pillar bump joints on (**a**) DPIG and (**b**) ENEPIG with various laser power densities and irradiation times.

Following the complete interconnection process, the surface finishes of the DPIG and ENEPIG were examined using an SEM, focusing on the cross-sections of the Cu/SnAg pillar bumps. Figure 9 illustrates these observations. At the DPIG and Cu/SnAg interface, the Cu_6_Sn_5_ intermetallic compound (IMC) was detected. During the reaction between the DPIG and SnAg, the thin Au and Pd layers dissolved into the molten solder, allowing the underlying Cu pad to react with the solder and form the Cu_6_Sn_5_ IMC [17]. As the laser power density and laser irradiation time increased, the thickness of the interface IMC tended to slightly increase, but there was no significant difference in thickness depending on the change in conditions. Filler traps, which occur when silica filler particles of non-conductive paste (NCP) are caught in bumps at the interface, were also observed under the conditions of 2.65 W/mm^2^–0.7 s and 2.65 W/mm^2^–1.0 s. Meanwhile, the commonly found Ni_3_Sn_4_ IMC was formed at the ENEPIG and Cu/SnAg pillar bump interface [18]. It was observed that the ENEPIG surface finish had thinner IMC growth than the DPIG surface finish because it contained an Ni layer that inhibited IMC growth. Additionally, elongated Ag_3_Sn IMCs were also found under conditions of high laser power density and long irradiation time.

**Figure 10:**
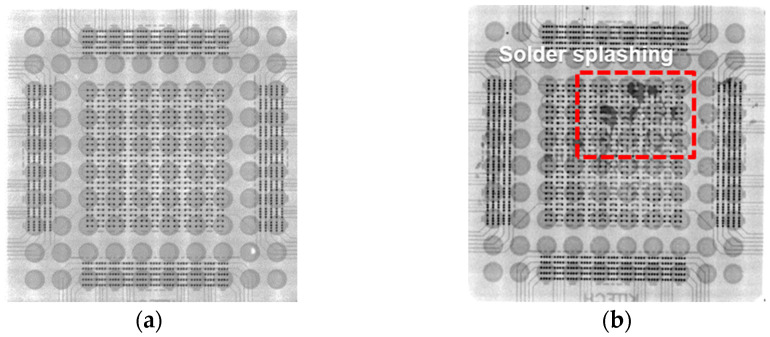
X-ray image of the SnAg/Cu pillar bump joints with a laser power density of 2.8 W/mm^2^. The laser irradiation time conditions were (**a**) 1.0 s and (**b**) 1.9 s.

A notable observation in Figure 9 is that for both the DPIG and ENEPIG samples, it was observed that some or all of the SnAg solder disappeared in the samples under conditions of high laser power density and long irradiation time. For both the DPIG and ENEPIG surface finishes, solder spreading was observed starting from the laser power density of 2.8 W/mm^2^, 1.3 s. To determine where the solder moved, samples with short (1.0 s) and long (1.9 s) laser irradiation times were observed by X-ray under the same laser power density conditions (2.8 W/mm^2^), as shown in Figure 10. Through X-ray imaging, we were able to observe the pad area and wiring of the chip and board. The small dot represents the part where the pillar bump of the chip and the board pad are joined, and the arranged circles indicate the solder ball pad part on the bottom of the board. When the laser irradiation time was 1.0 s, it was confirmed that the pillar bump on the chip side and the Cu pad on the substrate were properly aligned and well bonded. Meanwhile, as shown in Figure 10b, with a laser irradiation time of 1.9 s, dark spots were observed, as indicated by the square. The dark spots occur when the SnAg solder of the Cu/SnAg pillar bumps melts due to irradiation by a high-energy laser, causing the solder to splash, escape from the bump, and then re-coalesce and solidify.

**Figure 11:**
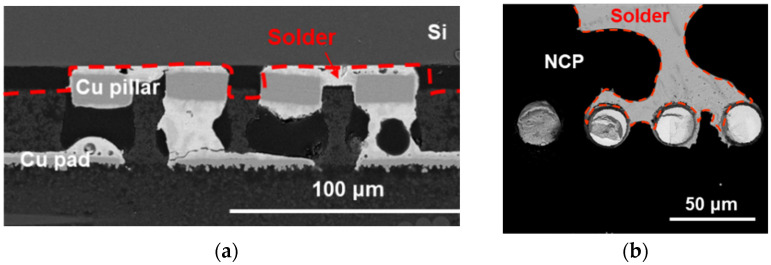
(**a**) Cross-sectional view and (**b**) top view image of solder splashing.

The cross-section and fracture surface of the sample joint where the solder disappeared were observed using an SEM and are shown in Figure 11. In the cross-sectional photo in Figure 11a, NCP is observed around the Cu/SnAg bump. Even if the solder disappears, the NCP remains intact, preserving the shape of the space where the solder was. Additionally, the interface between the Si chip and the Cu/SnAg pillar bump interface is separated, and the solder can be seen rising between them. Figure 11b is an SEM photo of the fracture surface after the shear strength test. The fracture occurred at the interface between the Si chip and the NCP, as indicated by the dotted line in Figure 11a, and it was observed that the solder spread between the Si chip and the NCP. In this study, we observed solder splashing occurring at high laser power density and analyzed its characteristics [19]. When solder is exposed to a laser for a long time or to high power for a short time, the temperature of the solder joint rises rapidly. In addition, the coefficient of thermal expansion (CTE) of Sn/Ag solder is relatively high, about 22.2 ppm/°C. Due to its high CTE, Sn/Ag solder cannot withstand high levels of laser power density over a short period of time, leading to a rapid increase in thermal stress. This causes the solder lose its original shape, disrupting the balance of surface tension and resulting in a phenomenon that spreads to the surrounding area. Since the Cu/SnAg is surrounded by NCP, the solder explodes, causing the interfaces between the Si chip, Cu/SnAg pillar, and NCP separate. The exploded molten solder escapes and splashes. Therefore, to suppress solder splashing defects, it is very important to optimize the laser power density and irradiation time.

**Figure 12:**
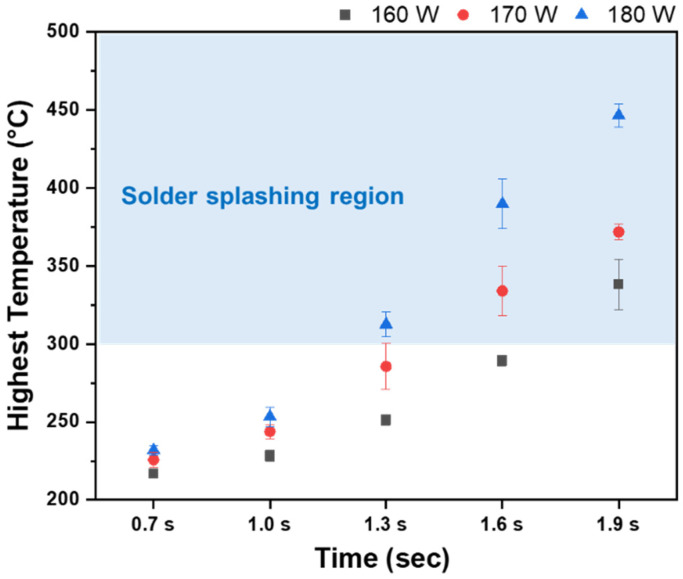
Highest temperature profile of the laser power density.

Figure 12 shows the temperature profile measured under various laser power densities using a laser pyrometer. Among the laser power densities, the lowest laser power density (2.5 W/mm^2^, 0.7 s) recorded a temperature of 221 °C, while the highest laser power density (2.8 W/mm^2^, 1.9 s) measured 444 °C. Additionally, solder splashing was observed for temperatures exceeding 300 °C, with a measurement of 301.8 °C under the conditions of 2.8 W/mm^2^ and 1.3 s.

### 3.2. Mechanical Properties with LAB Power Density

**Figure 13:**
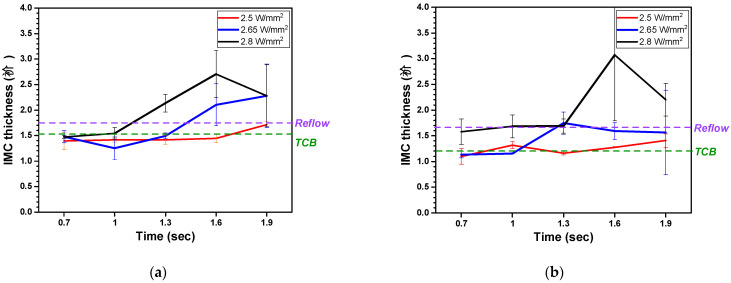
IMC thickness under LAB conditions for the (**a**) DPIG/SnAg and (**b**) ENEPIG/SnAg.

The IMC thickness according to laser power density is shown in Figure 13. The IMC thickness increased with the laser power density and irradiation time. At the DPIG’s highest temperature condition of 2.8 W/mm^2^, the IMC thickness was 2.3 μm, which was measured to be much thicker than the IMC thickness for MR and TCB [20]. The DPIG showed an overall higher IMC thickness compared to the ENEPIG. The high thickness of the DPIG is due to the absence of an Ni layer that acts as a diffusion barrier. When there was solder splashing, the IMC thickness relatively increased.

**Figure 14:**
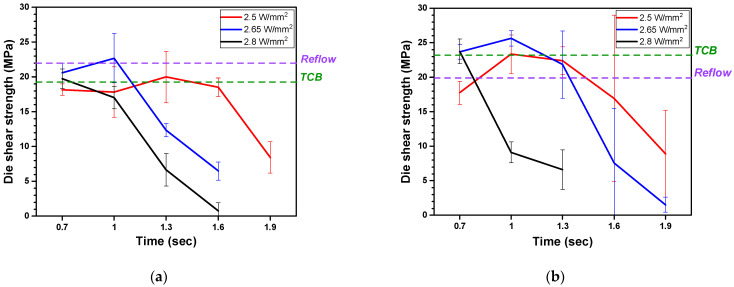
Graph of shear strength for (**a**) DPIG surface finishes and (**b**) ENEPIG surface finishes.

Figure 14 shows the die joint test results for the Cu/SnAg solder with an LAB joint and the DPIG and ENEPIG samples. The LAB samples showed higher shear strength compared to the MR and TCB samples. The shear strength was 22.69 MPa under the conditions of a laser power density of 170 W and an irradiation time of 1.0 s for the DPIG surface finish, and 25.67 Mpa for the ENEPIG surface finish sample. As the laser power density and irradiation time increased, the overall shear strength decreased and could not be measured under conditions where some solder spread.

**Figure 15:**
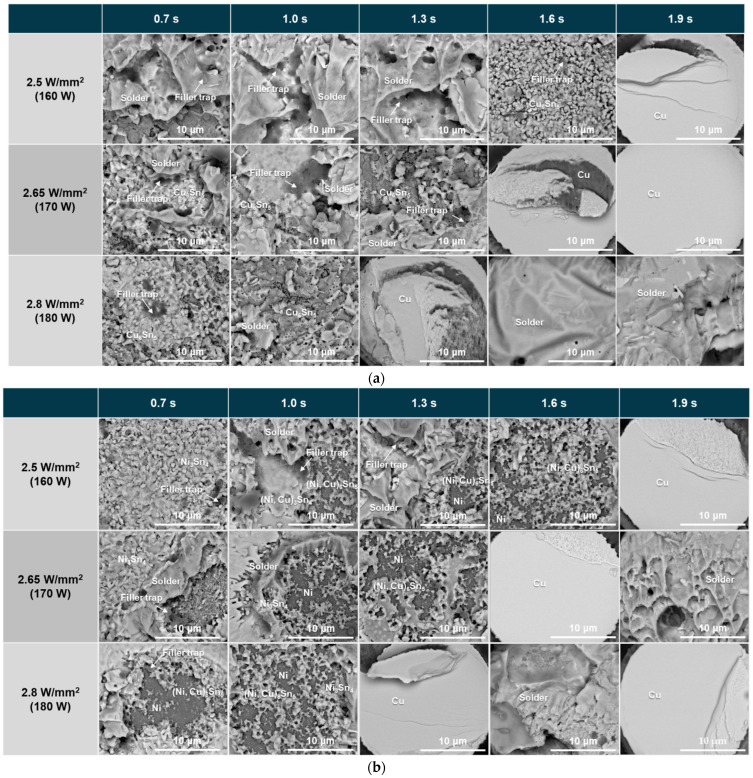
Fracture surfaces of the (**a**) DPIG/SnAg and (**b**) ENEPIG/SnAg pillar joints.

**Figure 16:**
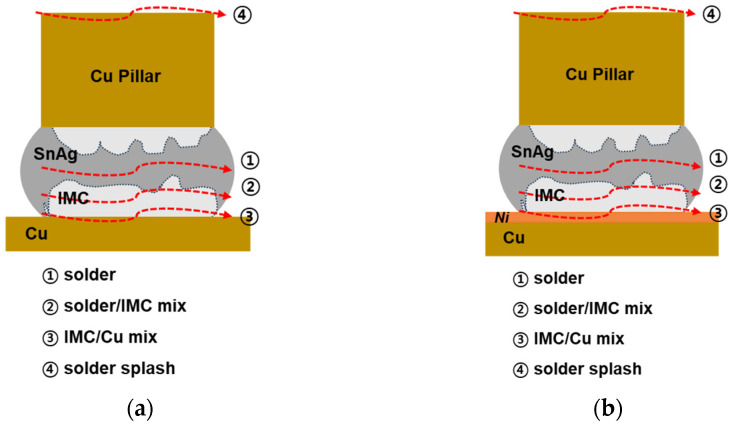
Schematic of the fracture modes: (**a**) DPIG/SnAg and (**b**) ENEPIG/SnAg.

After the die shear test, the fracture surface was analyzed using an SEM and is shown in Figure 15. The schematic diagram in Figure 16 shows the four fracture modes of the DPIG and Cu/SnAg pillar joints.

- Fracture mode 1: A fracture that occurs inside the SnAg solder;
- Fracture mode 2: A mixed fracture mode in the solder and Cu_6_Sn_5_ IMC;
- Fracture mode 3: A mixed fracture mode in the Cu_6_Sn_5_ IMC and Cu pad;
- Fracture mode 4: A fracture mode when solder splashing occurs.

The fracture surface image in Figure 15a shows that fracture occurred in a mixed mode in the solder and Cu_6_Sn_5_ IMC under low laser power density and short irradiation time conditions. As the power and time increased, a mode 3 fracture occurred in the Cu_6_Sn_5_ IMC and Cu pad, indicating that the fracture moved toward the Cu pad. Under high laser power density and long irradiation time conditions, solder splashing occurred, and fracture occurred at the interface between the Si and the Cu pillar where the solder had penetrated. Therefore, after the die shear test, the upper part of the Cu pillar and the solder spread were observed.

Figure 15b is an SEM image observing the fracture surface of the ENEPIG and Cu/SnAg pillar joint after the die shear test. Complex destruction occurred in the solder and IMC, or destruction occurred in the IMC and Ni, as shown in Figure 16. Similar to the DPIG sample, there were four fracture modes:

- Fracture mode 1: A fracture that occurs inside the SnAg solder;
- Fracture mode 2: A mixed fracture mode in the solder and Ni_3_Sn_4_ IMC;
- Fracture mode 3: A mixed fracture mode in the Ni3Sn4 IMC and Ni;
- Fracture mode 4: A fracture mode when solder splash occurs.

Unlike the DPIG sample, the ENEPIG had an Ni layer on the Cu pad, so fracture occurred in the Ni layer in the ENEPIG sample. As the laser power density and irradiation time increased, the fracture changed from SnAg–IMC mixed to IMC–Ni mixed mode, and as the laser power density and irradiation time increased further, solder splashing occurred and the upper part of the Cu pillar was observed.

**Figure 17:**
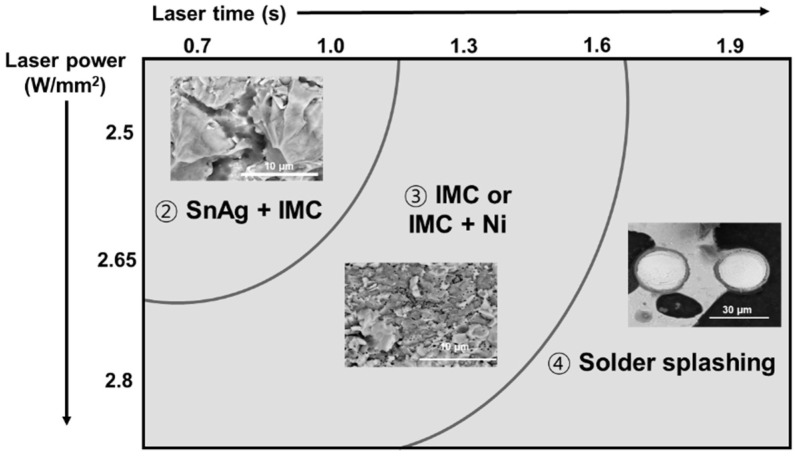
Fracture mode map based on the laser power density and irradiation time conditions.

Figure 17 is a fracture mode map according to the temperature and irradiation time. Under low temperature and irradiation time conditions, fractures occur within the solder and IMC. In the middle range, fracture occurs at the IMC–Cu or IMC–Ni interface. Under high temperature and irradiation time conditions, solder splashing occurs, and the solder that explodes out is at the interface between the Cu pillar and the Si wafer. Because this interface becomes the weakest part, fracture occurs at the interface between the Cu pillar and the Si. In the general MR process, the change in peak temperature is not large. However, in the case of laser bonding, the light absorption rate varies depending on the surface condition of the sample or the wiring design of the chip, resulting in many cases where the soldering temperature increases significantly in a short period of irradiation time. Therefore, unlike the general MR process temperature, the actual joint temperature increases significantly even with small changes in the laser power density or irradiation time, which causes the microstructure and physical properties of the joint to change significantly. In this study, even though the laser power density and irradiation time were very slightly increased, solder splashing, which was not seen in the existing MR or TCB, was discovered. This phenomenon depends on the laser power density and irradiation time of the laser, the surface condition of the sample, etc., in the actual LAB joining process. This means that these factors must be carefully considered. However, since LAB is a selective heating method that suppresses warpage, it is considered an appropriate method for addressing fine-pitch issues. Additionally, autonomous control using artificial intelligence is considered necessary to accurately control the laser processing conditions according to the sample.

## 4. Conclusions

This study investigated the bonding characteristics and mechanical properties of Cu/SnAg pillar bumps with Ni-less surface finishes using LAB. By comparing LAB with traditional bonding methods such as TCB and MR, we assessed the effectiveness of LAB in enhancing the bond quality and mechanical performance. Our findings highlight LAB’s potential as a highly effective method for fine-pitch semiconductor packaging. Our key findings are as follows:

1. High laser power density and irradiation time caused “solder splashing” where solder explosively vaporized and spread. This was particularly evident at 2.8 W/mm^2^ and 1.9 s, highlighting the need for precise laser parameter control.
2. The DPIG showed IMC thickness and higher shear strength (22.69 MPa) compared to the ENEPIG, which had a shear strength of 25.67 MPa. The Ni layer in the ENEPIG acted as a diffusion barrier, resulting in thinner IMC growth.
3. Mechanical performance: The LAB joints exhibited superior mechanical performance, with higher shear strength than the TCB and MR joints. The failure mechanisms shifted from solder to IMC-related fractures with increasing laser power density and irradiation time.
4. Fine-pitch applications: LAB minimized the thermal stress and warpage, making it highly suitable for fine-pitch applications and high-performance semiconductor packaging.

Academically, this research advances the understanding of LAB’s effectiveness and mechanisms, particularly in the context of Ni-less surface finishes. Industrially, it offers a promising alternative to traditional bonding techniques, potentially enhancing productivity and reliability in terms of semiconductor packaging. The phenomenon of solder splashing underscores the importance of precise parameter control in LAB, which could lead to further innovations and optimizations in bonding technologies. In future studies, reliability tests will be performed in various environments based on the LAB conditions optimized in this study to evaluate the long-term performance and durability, thereby enabling the development of more reliable processes.

## Data Availability

The original contributions presented in the study are included in the article, further inquiries can be directed to the corresponding authors.
